# The association between occupational factors, depression, and health-related quality of life in military women in the Republic of Korea: a cross-sectional study

**DOI:** 10.1186/s12955-021-01846-1

**Published:** 2021-09-14

**Authors:** Eunji Kwon, Jeongok Park, Sue Kim, Kyung Hee Lee

**Affiliations:** 1 College of Nursing, Yonsei University, Seoul, Republic of Korea; 2 Korea Armed Forces Nursing Academy, Daejeon, Republic of Korea; 3 Mo-Im Kim Nursing Research Institute and College of Nursing, Yonsei University, 50-1 Yonsei-ro, Seodaemun-gu, Seoul, 03722 Republic of Korea

**Keywords:** Military women, Health-related quality of life, Stress, Depression

## Abstract

**Background:**

Health-related quality of life (HRQOL) is an important concept to consider both individuals' ability to manage their daily lives and health status across the lifespan. Despite this variable's importance, there is a lack of clarification on the factors associated with HRQOL, especially for military women. The aim of this study was to examine factors associated with HRQOL of military women in the Republic of Korea (ROK) Army.

**Methods:**

This cross-sectional study included 196 participants who were currently within their 5-year service period. HRQOL was measured by the Korean version of the Short-Form 36 Health Survey Questionnaire version 2.0 (SF-36v2), and depression was assessed using the Korean version of the Patient Health Questionnaire-9 (PHQ-9). Differences in HRQOL according to general and occupational factors were analyzed using the independent t-test and analysis of variance (ANOVA). Multiple linear regression analysis was performed to identify factors associated with the HRQOL of women serving as military junior officers.

**Results:**

The mean score for the physical component summary (PCS) of SF-36v2 was 56.0 ± 5.8, and that for the mental component summary (MCS) of SF-36v2 was 47.2 ± 10.0. For depression, the mean score was 5.4 ± 5.2, whereas 19.4% of the participants scored more than 10 out of 27 points, which means moderate to severe. No variables showed statistically significant relationships with the PCS. However, military women showed a lower score for MCS when they were officers (adjusted *β* = − 3.52; 95% CI = − 5.47, − 1.58), had higher perceived stress (adjusted *β* = − 0.62, 95% CI = − 0.83, − 0.41), and a higher score for depression (adjusted *β* = − 0.86, 95% CI = − 1.10, − 0.63).

**Conclusions:**

Although depression levels were not severe, it was a significant factor of HRQOL. Stress and depression were found to be significant factors associated with the MCS in military women. Therefore, to improve their HRQOL, the ROK Army should provide early screening, intervention, and management program for high-risk military women. In addition, an appropriate organizational atmosphere within the military must be created to promote such programs.

## Introduction

Military women in the Republic of Korea (ROK) comprise officers and non-commissioned officers serving in the field through voluntary enlistment; the Ministry of National Defense stated that it planned to increase the proportion of female soldiers, who accounted for about 3.5% of all military officers in 2010, to 8.8% by 2020 [1]. In general, military junior officers are personnel with a service period of less than 5 years, and this group accounts for about 70% of military officers in their 20s and 30s [2]. This age group encompasses the transition from adolescence to adulthood and is among the healthiest periods in the life cycle [3]. This period is also characterized by extensive variation in individual health promotion activities, such as eating habits and physical activity, depending on the individual's personal choices [4], and in particular, since most female military junior officers are women of childbearing age, they are required to consider aspects of their reproductive health. Accordingly, the Ministry of National Defense is making efforts to create a healthy working environment and to establish policies for military women by identifying high-priority health problems and vulnerable groups through comprehensive studies of overall health conditions, medical institution use, and women's health [5].

As the definition of health has been extended from simply the absence of disease to the concept of complete well-being in recent years, researchers have taken an increasing interest in health-related quality of life (HRQOL), a concept that considers an individual’s ability to manage his or her daily life and a person’s health status across the lifespan [6, 7]. Measurements of HRQOL are important methodological tools for routine monitoring, especially when providing healthcare for vulnerable groups [8]. With the recent increase in interest in HRQOL, numerous studies have been conducted in diverse populations differing in terms of gender and age groups [6, 9, 10]. Those studies have shown that high self-efficacy, stress management, formation of a desirable eating attitude, and appropriate physical activity are important factors that improve HRQOL at the individual level [3, 6, 9].

For military women serving in the ROK Army, it has been confirmed that distinctive characteristics of the armed forces, such as rank, length of service, working hours, number of overtime hours, and working areas, are related to health-promoting activities [11]. These results suggest that both systematic health care policies at the organizational level and individual issues should be considered when formulating strategies for improving HRQOL of Korean military women [11]. In addition, since most Korean military women are in their childbearing age, specific attention should be paid to aspects of reproductive health [9, 11]. Therefore, for establishing strategies to improve the HRQOL of Korean military women, both individual-level health promotion activities and women’s normal health problems according to their life cycle should be considered in light of the distinctive features of the military environment. Nevertheless, there was a lack of research that explored the factors that affect soldiers’ HRQOL [10, 11], especially with appropriate consideration of both individual health promotion activities and women’s life cycle in the unique environment of the military.

**Fig. 1:**
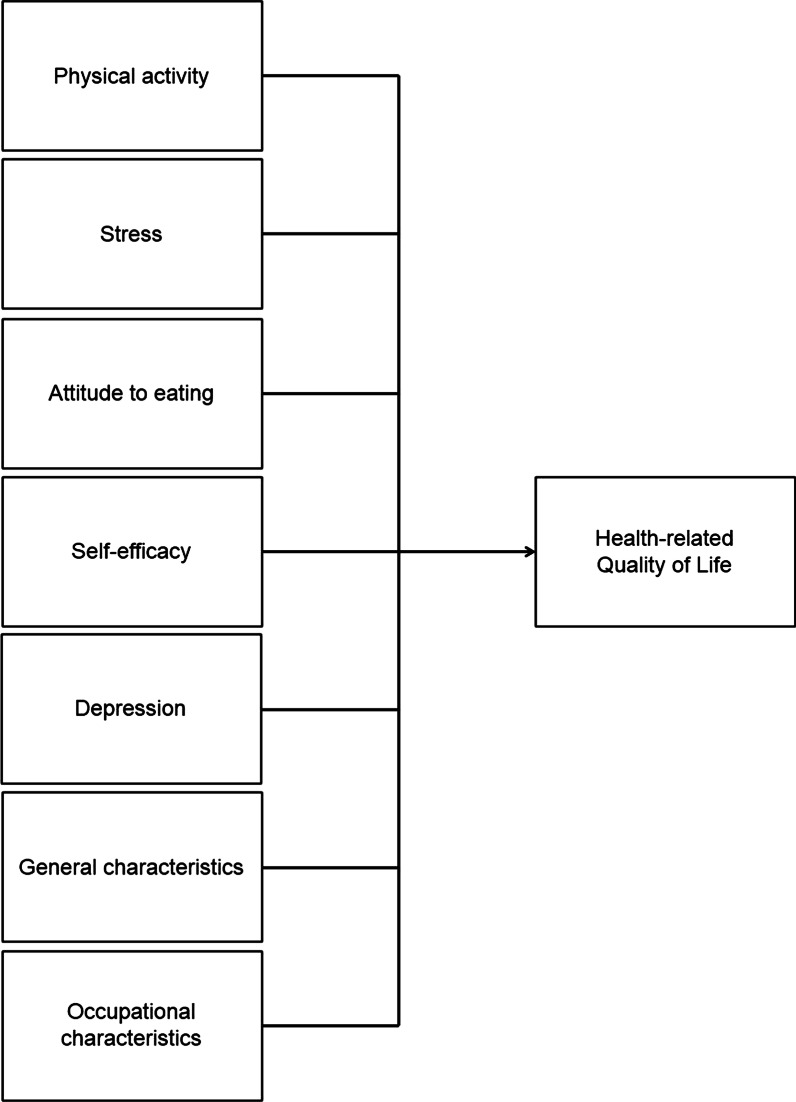
Conceptual framework

The conceptual framework for the current study was developed based on the comprehensive literature review (Fig. 1). The variables of physical activity, stress, attitude to eating, self-efficacy, and depression, as well as general and occupational characteristics such as age, education, marital status, age at menarche, delivery experience, rank, and branch were included in the conceptual framework to identify factors associated with the HRQOL. The purpose of this study was to explore the level of HRQOL and identify factors associated with HRQOL among military women serving in the ROK Army. Specifically, we hypothesized that military women’s physical activity, stress, attitude to eating, self-efficacy, depression, and occupational characteristics will be significantly associated with their HRQOL.

## Materials and methods

### Study design and sample

**Fig. 2:**
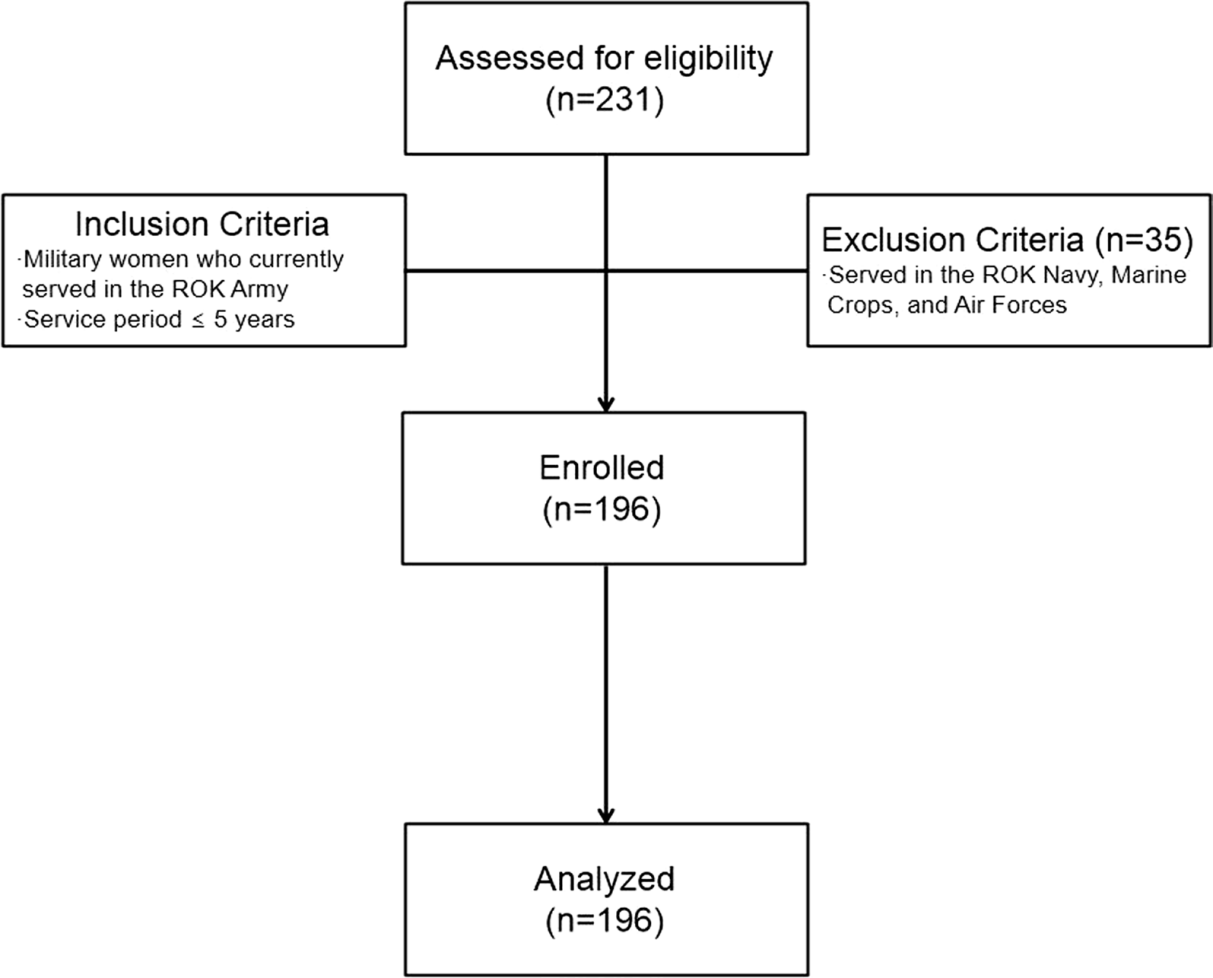
Flow-chart of this study

This cross-sectional study was conducted in November 2019. The inclusion criteria were military women who currently served in the ROK Army with a service period within 5 years and voluntarily chose to participate in this study after receiving an explanation of the purposes and goals of the research. The exclusion criteria were military women who currently served in the ROK Navy, Marine Corps, and Air Forces because their working area and occupational environment were different from those of the Army. The sample size was calculated using G*Power 3.1.9.7. To perform a multiple linear regression analysis, the sample size with a significance level of 0.05, power of 0.80, effect size (medium) of 0.15, and 22 independent variables was calculated as at least 163 and, after considering the attrition rate of 20%, was calculated as 196 [12]. A total of 231 military women initially accessed the online website to participate in the study, but 35 women were excluded because they did not meet the inclusion criteria (Fig. 2). Therefore, 196 women who completed the survey were included for the data analysis. The flow-chart for sample recruitment was illustrated in Fig. 2.

Because military women work throughout the nation and the proportion of military women in each division is only approximately 5%, the survey was conducted in an online and mobile format. By cooperating with official announcements and text messages, we encouraged participation in the survey. The recruitment notice included a description of the purpose and methods of this study, the conditions for participants, the benefits and risks of this study, the discontinuation of voluntary participation, and the assurance of anonymity for the study participants.

Those who fulfilled the inclusion criteria and voluntarily agreed to participate in the study were provided a link for the online survey. Screening questions were also presented on the first screen of the online survey to ensure that participants met the selection criteria. If all criteria were met, the participant completed the survey after viewing and an explanation stating that completing the survey was considered as constitute agreement to participate in this study. The participants who completed the survey received approximately $3(USD) gift cards.

The average response time for this survey was about 15 min. In accordance with institutional review board (IRB) approval (IRB approval number: Y-2019-0162), a protocol was put in place to ensure that the researcher was notified of responses anonymously and that the respondents’ phone numbers were not transmitted to the researcher. In order to prevent duplicate responses from the same participants and to ensure that the survey was not distributed beyond the intended participants, researchers monitored survey participation twice a day.

### Measurement

The research instrument was a self-reported survey questionnaire that consisted of 116 questions, including 36 questions about HRQOL, 7 questions about physical activity, 10 questions about stress, 26 questions about attitudes to eating, 10 questions about self-efficacy, 9 questions about depression, and 18 questions about general and occupational characteristics. The original authors approved the use of the relevant tools, the validity and reliability of which have been demonstrated in previous studies.

### Dependent variables

#### Health-related quality of life

HRQOL was the dependent variable of this study. The Korean version of the Short-Form 36 Health Survey Questionnaire version 2.0 (SF-36v2) is a self-evaluation scale used to measure HRQOL [13], and consists of 36 items that measure eight health domains: physical function, role limitations due to physical problems, bodily pain, general health perception, vitality, social functioning, role limitations related to emotional problems, and general mental health. The score for each domain ranges from 0 to 100, with higher scores indicating better HRQOL. These domains can be categorized into the physical component summary (PCS) and mental component summary (MCS). PCS and MCS scores are represented on a standardized scale (as a T score with a mean of 50 and standard deviation (SD) of 10). The internal consistency of the PCS and MCS was quantified using Cronbach’s α, with values of 0.78 and 0.60, respectively.

### Independent variables

#### Physical activity

The Korean version of the International Physical Activity Questionnaire (IPAQ) was used to estimate participants’ level of physical activity during the previous 7 days [14]. The items of the IPAQ are structured to provide a domain-specific score for walking, moderate-intensity, and vigorous-intensity activity. The results are presented as the estimated energy expenditure in metabolic equivalent-minutes per week (MET hours/week). The number of MET hours/week for a specific activity is computed by multiplying the MET value for the activity (3.3 for walking, 4.0 for moderate-intensity activity, and 8.0 for vigorous-intensity activity) by the number of hours spent on that activity [14].

#### Stress

The Korean version of the Perceived Stress Scale (PSS) was applied to assess the degree to which respondents perceived their lives to be unpredictable and uncontrollable over the past month [15]. The PSS consists of 10 items, and higher scores indicate more severe stress. The internal consistency of the questionnaire was confirmed by a Cronbach’s α of 0.88 in this study.

#### Attitude to eating

Disordered eating in participants was assessed using the Korean version of Eating Attitudes Test-26 (EAT-26) questionnaire. The EAT-26 questionnaire includes 26 items in 4 domains: (1) self-control of eating and bulimic symptom, (2) preoccupation with being thinner, (3) food preoccupation, and (4) dieting [16, 17]. Each item is responded to on a 6-point Likert scale, but not all positions on the scale are scored. Each item is given a score of zero for ‘sometimes,’ ‘rarely,’ and ‘never’; a score of 1 for ‘often’; a score of 2 for ‘usually’; and a score of 3 for ‘always.’ The total score ranges from 0 to 78, with higher scores indicating that a respondent is at a higher risk of eating disorders. The internal consistency of the questionnaire was shown by a Cronbach’s α of 0.87 in this study.

#### Self-efficacy

The Korean adaptation of the General Self-Efficacy Scale was used to measure self-efficacy [18]. It consists of 10 items concerning self-confidence and is measured on a 4-point Likert scale. Each question was answered with scores from ‘not at all true’ (1) to ‘exactly true’ (4). The total score ranges from 10 to 40, with higher scores indicating better self-efficacy. The internal consistency of the questionnaire was demonstrated by a Cronbach’s α of 0.89 in this study.

#### Depression

The Korean version of the Patient Health Questionnaire-9 (PHQ-9) was applied to assess the degree of depression [19]. PHQ-9 is a self-evaluation scale used to measure mental health at primary health care centers [20]. It consists of 9 items assessing the frequency with which patients have experienced depressive thoughts or feelings over the prior 2 weeks. The severity of depressive disorder is considered mild for scores ranging from 5 to 9 and moderate to severe for score from 10 or more [19]. The internal consistency of the questionnaire was shown by a Cronbach’s α of 0.90 in this study.

#### General and occupational characteristics

The following general characteristics were collected: age, body mass index (BMI), religion, education level, marital status, living with spouse, age at menarche, regularity of the menstrual cycle, length of the menstrual cycle, pregnancy and delivery experience, and history of oral contraception use. BMI classification used for Asian populations [21]. The occupational characteristics were rank, branch, duration of service, working area, duty time, service type, and number of overtime days.

### Data analysis

The collected data were statistically processed using the SPSS version 25 (IBM Corp., Armonk, NY, USA). Descriptive statistics were calculated for participants’ characteristics. The differences in HRQOL were analyzed using the independent t-test and analysis of variance (ANOVA). Multiple linear regression analysis was conducted to identify factors significantly associated with HRQOL among military women. A two-tailed probability value of *p* < 0.05 was considered to indicate statistical significance.

## Results

### Differences in health-related quality of life according to participants’ general and occupational characteristics

Table 1 presents participants’ general and occupational characteristics. The mean ± SD age of the participants was 25.2 ± 2.2 years, and their mean ± SD BMI was 21.8 ± 2.3 kg/m^2^, with 145 (74.0%) participants having a BMI in the normal range (18.5 to 23.0 kg/m^2^) [21]. 24 (12.2%) women were married, and 16 (66.7%) of them lived with their spouses or children. All of the participants responded that they currently had menstrual cycles and 48 (26.5%) indicated that they experienced menstrual irregularity. Approximately one-quarter of participants had taken oral contraceptive pills (25.5%). Moreover, on average, oral contraceptive pills were taken for 5.1 ± 6.7 months. The most common reason for taking oral contraceptive pills was for menstrual suppression during dispatch and training (42.0%), followed by temporary contraception (32.0%) and therapeutic purposes for a disease (26.0%). There were 69 (35.2%) non-commissioned officers and 127 (64.8%) officers, distributed among combat, technical and administrative, and specialized branches (39.3%, 20.9%, and 39.8%, respectively). Average overtime per month was 7.7 ± 8.2 days.

**Table 1:** Differences in health-related quality of life according to general and occupational characteristics (N = 196)

Variable	Categories	n (%) or Mean ± SD	Health-related quality of life			
			Physical component summary	t or F or r (*p*)	Mental component summary	t or F or r (*p*)
			Mean ± SD		Mean ± SD	
General characteristics						
Age (years)		25.2 ± 2.2		0.05 (0.519)		0.14 (0.056)
Body mass index (kg/m^2^)	< 18.5	7 (3.6)	59.8 ± 2.8	2.68 **(0.048)**^†^	46.0 ± 8.2	0.07 (0.978)
	18.5–22.9	145 (74.0)	55.8 ± 5.6		47.3 ± 9.7	
	23.0–24.9	21 (10.7)	57.7 ± 4.3		46.6 ± 11.3	
	≥ 25	23 (11.7)	54.0 ± 7.6		47.4 ± 11.9	
Religion	Yes	101 (51.5)	55.4 ± 5.8	1.33 (0.184)	47.2 ± 9.9	− 0.13 (0.896)
	No	95 (48.5)	56.5 ± 5.7		47.1 ± 10.3	
Education	≤ High school	9 (4.5)	58.2 ± 6.1	1.16 (0.316)	45.3 ± 10.0	0.43 (0.648)
	College	45 (23.0)	55.2 ± 5.5		48.2 ± 10.0	
	≥ University	142 (72.5)	56.0 ± 5.8		46.9 ± 10.1	
Marital status	Unmarried	172 (87.8)	55.8 ± 5.9	− 1.06 (0.290)	46.6 ± 9.7	− 2.22 **(0.027)**
	Married	24 (12.2)	57.1 ± 4.7		51.4 ± 11.6	
Living situation (n = 24)	With spouse	16 (66.7)	58.6 ± 2.9	− 1.94 (0.086)	56.4 ± 4.6	− 2.77 **(0.025)**
	Without spouse	8 (33.3)	54.1 ± 6.2		41.3 ± 15.0	
Age at menarche (years)		13.2 ± 1.5		− 0.05 (0.476)		0.01 (0.914)
Menstrual cycle (n = 181)	Regular	133 (73.5)	56.3 ± 5.6	0.98 (0.327)	47.2 ± 10.6	0.14 (0.891)
	Irregular	48 (26.5)	55.4 ± 6.1		47.0 ± 9.2	
Length of menstrual cycle (n = 181)		34.6 ± 26.8		0.06 (0.399)		0.01 (0.914)
Pregnancy experience	Yes	10 (5.1)	56.4 ± 5.0	− 0.25 (0.806)	51.4 ± 8.7	− 1.38 (0.169)
	No	186 (94.9)	55.9 ± 5.8		46.9 ± 10.1	
Number of pregnancies (n = 10)	1	7 (70.0)	56.6 ± 5.5	0.20 (0.849)	48.4 ± 8.8	− 2.99 **(0.023)**
	2 or more	3 (30.0)	55.9 ± 4.4		58.5 ± 0.8	
Delivery experience	Yes	8 (4.1)	56.1 ± 5.4	− 0.08 (0.933)	52.5 ± 9.4	− 1.53 (0.127)
	No	188 (95.9)	55.9 ± 5.8		46.9 ± 10.0	
Age at first birth (n = 8)		26.5 ± 2.5		− 0.20 (0.638)		− 0.58 (0.134)
History of oral contraceptive use	Yes	50 (25.5)	55.4 ± 5.8	0.81 (0.419)	48.6 ± 9.6	− 1.15 (0.251)
	No	146 (74.5)	56.1 ± 5.8		46.7 ± 10.2	
Duration of oral contraceptive use (month)		5.1 ± 6.7		− 0.04 (0.669)		− 0.15 (0.159)
Occupational characteristics						
Rank	Non-commissioned officer	69 (35.2)	55.5 ± 6.1	− 0.88 (0.382)	49.4 ± 9.6	2.39 **(0.018)**
	Officer	127 (64.8)	56.2 ± 5.6		45.9 ± 10.1	
Branch	Combat	77 (39.3)	56.1 ± 5.8	0.19 (0.830)	47.4 ± 10.4	0.05 (0.949)
	Technical & Administrative	41 (20.9)	55.5 ± 6.0		47.2 ± 10.6	
	Specialized	78 (39.8)	56.0 ± 5.7		46.9 ± 9.4	
Duration of service (month)		32.7 ± 15.7		0.10 (0.171)		0.12 (0.088)
Working area	Urban area	94 (48.0)	56.2 ± 4.8	0.56 (0.576)	48.0 ± 9.2	1.17 (0.243)
	Rural area	102 (52.0)	55.7 ± 6.6		46.4 ± 10.7	
Duty time	Day time	164 (83.7)	56.2 ± 5.7	0.82 (0.444)	47.2 ± 10.2	1.03 (0.358)
	Night time	9 (4.6)	54.7 ± 7.4		42.9 ± 12.6	
	Shift work	23 (11.7)	54.8 ± 5.8		48.5 ± 7.5	
Service type	Short-term service	80 (40.8)	55.4 ± 6.4	0.96 (0.385)	46.6 ± 9.9	0.23 (0.793)
	Extended service	44 (22.5)	55.8 ± 5.4		47.5 ± 10.4	
	Long-term service	72 (36.7)	56.7 ± 5.3		47.6 ± 10.0	
Number of overtime days		7.7 ± 8.2		− 0.06 (0.391)		− 0.16 **(0.023)**

^†^Multiple comparisons with Bonferroni procedures resulted in no significant differences between groups

In this study, PCS and MCS scores are represented on a standardized scale (as a T score with a mean of 50 and SD of 10). Significant differences were found in the PCS score according to BMI (F = 2.68, *p* = 0.048). However, multiple comparisons with Bonferroni procedures resulted in no significant differences between groups. The married military women reported significantly higher MCS scores than the unmarried military women (51.4 and 46.6, respectively, *p* = 0.027). Among the married military women, the MCS score was significantly different according to whether respondents lived with their spouses (t = − 2.77, *p* = 0.025). Significant differences in the MCS score were found according to rank, with officers reporting significantly lower scores than non-commissioned officers (45.9 and 49.4, respectively, *p* = 0.018). A higher number of overtime days per month was associated with a lower MCS score (r = − 0.16, *p* = 0.023).

### Descriptive statistics for research variables

Table 2 presents descriptive statistics for HRQOL and the other variables evaluated in the questionnaire. The scores of the two main domains, PCS and MCS, were 56.0 ± 5.8 and 47.2 ± 10.0, respectively. Each domain was converted to a score of 0–100 points, with higher scores indicating better HRQOL. The highest score was reported for the physical function domain (92.2 ± 14.2) and the lowest score for vitality (58.5 ± 20.2).

**Table 2:** Descriptive statistics of the research variables (N = 196)

Variables	Categories	Possible range	n (%) or Mean ± SD
Health-related quality of life			
PCS			56.0 ± 5.8
	Physical function	0–100	92.2 ± 14.2
	Role limitations due to physical problems	0–100	88.5 ± 16.6
	Bodily pain	0–100	80.8 ± 20.3
	General health perception	0–100	73.5 ± 19.0
MCS			47.2 ± 10.0
	Vitality	0–100	58.5 ± 20.2
	Social functioning	0–100	84.2 ± 19.2
	Role limitations due to emotional problems	0–100	83.0 ± 21.8
	General mental health	0–100	69.5 ± 19.6
Physical activity	Category 1 (inactive)		37 (18.9)
	Category 2 (minimal physical activity)		96 (49.0)
	Category 3 (health-enhancing physical activity)		63 (32.1)
MET (min/week)	Total physical activity		2915.5 ± 3431.0
	Walking activity		1460.4 ± 2373.0
	Moderate activity		495.1 ± 813.1
	Vigorous activity		960.0 ± 1226.0
	Sitting activity per day (min)		370.3 ± 250.8
Perceived stress		0–40	18.0 ± 6.2
Attitudes to eating		0–78	9.4 ± 9.3
	Self-control of eating and bulimic symptom		2.4 ± 3.9
	Preoccupation with being thinner		4.1 ± 4.0
	Food preoccupation		0.9 ± 1.5
	Dieting		1.9 ± 2.7
Self-efficacy		10–40	29.0 ± 4.2
Depression		0–27	5.4 ± 5.2
	0–4 (Normal)		106 (54.1)
	5–9 (Mild depression)		52 (26.5)
	10 ≤ (Moderate to severe depression)		38 (19.4)

PCS: physical component summary, MCS: mental component summary, MET: metabolic equivalent

When the participants were categorized according to the criteria for IPAQ, 37 (18.9%) participants were inactive, 96 (49.0%) were minimally physical active, and 63 (32.1%) engaged in a health-enhancing level of physical activity. The mean scores for perceived stress, attitudes to eating, and self-efficacy were 18.0 ± 6.2, 9.4 ± 9.3, and 29.0 ± 4.2, respectively. The mean score of depression was 5.4 ± 5.2, and 38 (19.4%) of the participants had a total score of more than 10 (moderate to severe).

### Factors associated with health-related quality of life

Table 3 presents the results of multiple linear regression to identify factors associated with the participants’ HRQOL. An independent t-test and one-way ANOVA were performed to confirm statistically significant variables of HRQOL among the general and occupational characteristics (Table 1). Among the significant variables, living situation and number of pregnancies were not included in the multiple linear regression model because whole participants did not respond to those questions. Furthermore, the variables suggested in the conceptual framework such as physical activity, stress, attitude to eating, self-efficacy, and depression were included in the multiple linear regression model, as previous studies found an association with HRQOL. The multicollinearity between the independent variables that were included in the multiple linear regression was examined. As a result, the overall variance inflation factor was less than 10, confirming the independence between independent variables.

**Table 3:** Factors associated with health-related quality of life (N = 196)

Variable	Health-related quality of life			
	Physical Component Summary		Mental Component Summary	
	Crude *β* [95% CI]	Adjusted *β* [95% CI]	Crude *β* [95% CI]	Adjusted *β* [95% CI]
General and occupational characteristics				
Marital status (reference = unmarried)	1.33 [− 1.15, 3.82]	0.68 [− 1.83, 3.19]	4.82 [0.55, 9.09]	0.71 [− 2.09, 3.51]
Military rank (reference = non-commissioned officer)	0.76 [− 0.95, 2.46]	1.20 [− 0.54, 2.95]	− 3.55 [− 6.47, − 0.62]	− 3.52 [− 5.47, − 1.58]
Number of overtime days	− 0.04 [− 0.14, 0.06]	− 0.05 [− 0.16, 0.05]	− 0.20 [− 0.37, − 0.03]	− 0.03 [− 0.15, 0.08]
Physical activity (reference = Category 2; minimal physical activity)				
Category 1 (inactive)	− 0.85 [− 2.93, 1.23]	− 0.29 [− 2.50, 1.93]	− 2.28 [− 5.89, 1.33]	− 0.85 [− 3.32, 1.62]
Category 3 (health-enhancing physical activity)	0.74 [− 1.00, 2.49]	1.35 [− 0.57, 3.27]	0.19 [− 2.85, 3.22]	0.37 [− 1.77, 2.51]
Perceived stress	− 0.21 [− 0.34, − 0.08]	− 0.09 [− 0.28, 0.09]	− 1.11 [− 1.28, − 0.95]	− 0.62 [− 0.83, − 0.41]
Attitudes to eating	− 0.11 [− 0.19, − 0.02]	− 0.08 [− 0.18, 0.02]	− 0.33 [− 0.48, − 0.19]	− 0.02 [− 0.13, 0.09]
Self-efficacy	0.25 [0.05, 0.44]	0.07 [− 0.15, 0.30]	0.80 [0.48, 1.12]	− 0.02 [− 0.27, 0.23]
Depression	− 0.23 [− 0.39, − 0.08]	− 0.08 [− 0.29, 0.13]	− 1.37 [− 1.56, − 1.18]	− 0.86 [− 1.10, − 0.63]
R^2^	–	0.091	–	0.625
Adjusted R^2^	–	0.047	–	0.607
F (*p*)	–	2.07 (0.035)	–	34.48 (<0 .001)

95% CI: 95% Confidence interval

The values of R^2^ and adjusted R^2^, which indicate the goodness of fit of the model [22], were 0.091 and 0.047 for PCS and 0.625 and 0.607 for MCS, respectively. No factors had a statistically significant effect on PCS scores, while rank, perceived stress, and depression were all statistically significantly associated with MCS scores.

The average MCS score of officers was 3.52 lower than that of non-commissioned officers (adjusted *β* = − 3.52; 95% CI = − 5.47, − 1.58). Higher levels of perceived stress were associated with lower MCS scores (adjusted *β* = − 0.62, 95% CI = − 0.83, − 0.41); each 1-unit increase in stress led to a 0.62-point decrease in the MCS score after controlling for other variables (*p* < 0.001). And higher levels of depression were associated with lower MCS scores (adjusted *β* = − 0.86, 95% CI = − 1.10, − 0.63).

## Discussion

The findings of this study provide important information about HRQOL among military women serving in the ROK Army. Approximately 7,550 military women currently serve in the ROK Army [23]. Although the number of military women in the ROK is increasing, research on them remains insufficient [5]. Furthermore, limited research has focused exclusively on particular rank or branch; therefore, this study is meaningful in that it provides basic data for understanding HRQOL among military women in the Army, who account for 70% of the ROK whole military women [5].

The current study showed that the PCS score for military women was 56.0 points, which is higher than the score of 50.7 points reported for the ROK female undergraduates of the same age and the score of 53.5 points reported for the US military women of the same age [9, 24]. In previous studies, PCS was associated with being married, higher educational attainment, and higher military rank among US military women [24], and lower BMI and higher exercise frequency among Croatian university students [25]. However, no variables significantly associated with the PCS in this study. This is most likely because military women in the ROK Army who participated in the current study engage in individual health promotion behaviors through regular physical training and medical check-ups every year and receive systematic health management.

The average MCS score was 47.2 points; although this value is higher than the 45.3 points reported for the ROK female undergraduates [9], it is lower than 51.0 points for the US military women of the same age [24]. Regarding the factors associated with MCS, officers had a significantly lower score for MCS than non-commissioned officers. This may reflect the differences in duty characteristics between officers and non-commissioned officers [26]. In addition, officers are promoted through a pyramid structure, which poses difficulties in terms of competitiveness, a discriminatory retirement age, a lack of job security, and individual military professional development [27].

Stress and depression were also known to have a negative effect on MCS, as this study has confirmed [26, 28]. Therefore, further research is needed to improve the mental aspect of HRQOL among military women in the ROK Army. In a previous study [26], the quality of life of married military women was lower when they were not living with their families, had no support system, or experienced high stress in the workplace. These results suggest that working conditions need to be addressed in order to improve HRQOL among military women in the ROK Army. Currently, the ROK Army operates a joint childcare center to support work–family balance and strives to resolve psychological conflicts that cause stress and depression for military women by securing replacement personnel for those who have taken leave [23]. Regarding the negative effect of depression on the MCS in the current study, this result is consistent with previous studies [29]. In the US military, based on the National Defense Authorization Act, which has been strengthened since 2012, the Army has been required to perform mandatory screening tests for all soldiers each year through existing regular medical check-ups [30]. Considering that twice as many female officers are depressed as civilians of the same age, and that female officers have higher depression scores than male officers [29, 31], it should be a priority to identify vulnerable groups through depression screening tests. To manage high-risk groups, the ROK Army needs to pay attention to the identification and systematic management of depression where indicated.

In conclusion, the ROK Army should continue to seek various ways to improve the MCS in order to resolve the mental difficulties faced by military women. There is currently no separate mental health program for military women in the ROK Army. Existing mental health programs focus on post-traumatic stress disorder [32], as such, insufficient research and interventions have been implemented to improve the MCS as part of HRQOL. Furthermore, different factors influence quality of life between male soldiers and female officers, including social support, adjustment to military service, physical environment, and health behaviors [33].

Our research has the following limitations. First, it is difficult to determine causality because this study is based on cross-sectional data obtained from a survey. Reporting bias may have been present because all variables were measured by self-report questionnaires from respondents. Second, because the participants were rewarded with gift cards, these findings may cause extrinsic incentives bias. Third, because this study adopted a convenience sampling method and one of the inclusion criteria was only women who had served in the military for less than 5 years, the participants in the current study were not representative of the ROK Army women. Therefore, the results of this study should be interpreted with caution. Finally, future studies that can validate a theoretical framework could further confirm the variables and their relationships.

Despite these limitations, this study has the following strengths. First, in contrast to previous studies conducted mainly among male soldiers, this study assessed HRQOL and analyzed the factors that influenced it among military women in the ROK Army. Second, occupational and female-specific characteristics were considered in addition to general characteristics. For women, it is meaningful to consider these factors because psychological well-being is closely related with employment, marriage, pregnancy, and childcare, necessitating individualized research and interventions [34]. Third, we identified factors with negative and positive effects on the MCS. These findings are helpful in identifying which interventions should be implemented to improve the MCS.

## Conclusion

This study was conducted to identify factors associated with HRQOL among military women in the ROK Army. Stress and depression were found to be significant factors associated with the MCS. Therefore, we suggest that the ROK Army should provide early screening, intervention, and management for groups of high-risk military women. First, programs are needed to identify and manage risk factors that cause stress, and an appropriate organizational atmosphere within the military must be created to promote such training programs. Second, depression screening tests should be required for early management by classifying individuals at a high-risk of depression as a vulnerable group. Finally, given the high proportion of depression, it is suggested that regular HRQOL surveys are needed for members of the ROK Army, including military women.

## Abbreviations

CI: Confidence interval
ROK: Republic of Korea
HRQOL: Health-related quality of life
IRB: Institutional review board
SF-36v2: Short-Form 36 Health Survey Questionnaire version 2.0
PCS: Physical component summary
MCS: Mental component summary
SD: Standard deviation
IPAQ: International Physical Activity Questionnaire
MET: Metabolic equivalent
PSS: Perceived Stress Scale
EAT-26: Eating Attitudes Test-26
PHQ-9: Patient Health Questionnaire-9
BMI: Body mass index
ANOVA: Analysis of variance

## Declarations

### Availability of data and materials

All data generated or The data supporting the findings of this study are not publicly available due to security of the ROK army.
